# Identification of pyrimidine metabolism-based molecular subtypes and prognostic signature to predict immune landscape and guide clinical treatment in prostate cancer

**DOI:** 10.1080/07853890.2025.2449584

**Published:** 2025-01-13

**Authors:** Yu-Zhong Yu, Xiao Xie, Mao-Ping Cai, Ya-Ying Hong, Yang-Zi Ren, Xi Kang, Hai-Chen Yan, Yang Xiong, Hong Chen, Xing-Cheng Wu, Dao-Sheng Luo, Shan-Chao Zhao

**Affiliations:** a Department of Urology, The Third Affiliated Hospital of Southern Medical University, Guangzhou, China; b Department of Urology, Nanfang Hospital, Southern Medical University, Guangzhou, China; c Department of Urology, Pingxiang People’s Hospital, Pingxiang, China; d Department of Oncology, The First Affiliated Hospital of Guangzhou University of Chinese Medicine, Guangzhou, China; e Luoyang Key Laboratory of Organic Functional Molecules, College of Food and Drug, Luoyang Normal University, Luoyang, China; f Department of Urology, Peking Union Medical College Hospital, Peking Union Medical College, Chinese Academy of Medical Sciences, Beijing, China; g Department of Urology, The Tenth Affiliated Hospital of Southern Medical University (Dongguan People’s Hospital), Dongguan, China; h Department of Urology, The Fifth Affiliated Hospital, Southern Medical University, Guangzhou, China

**Keywords:** Pyrimidine metabolism, prognostic signature, immunity, thymidine kinase 1, prostate cancer

## Abstract

**Background:**

We previously described the enrichment of plasma exosome metabolites in CRPC, PCa, and TFC cohorts, and found significant differences in pyrimidine metabolites. The PMGs is associated with the clinical prognosis of several cancers, but its biological role in PCa is still unclear.

**Methods:**

This study extracted 98 reliable PMGs, and analyzed their somatic mutations, expression levels, and prognostic significance. Unsupervised clustering was applied to classify patients with PCa into clusters based on six PMGs that were related to the prognosis of PCa. The TME, gene mutations, and immune escape ability were compared among the clusters. A scoring algorithm based on prognostic PMGs, referred to as the PMGscore, was developed. TK1 was identified and the biological functions of TK1 were determined using loss-of-function experiments. RNA sequencing was subsequently performed to determine the molecules associated with the underlying mechanisms of TK1 function.

**Results:**

In total, six out of 98 PMGs simultaneously exhibited differential expression in PCa and were correlated with BCR. Patients were clustered into two clusters according to the expression levels of these six PMGs, which reflected distinct clinical outcomes and immune cell infiltration characteristics. Clinical features, tumor prognosis, and functional annotation were analyzed. Subsequently, we constructed a prognostic signature using these six PMGs. In combination with other clinical traits, we found that the six PMGs’ prognostic signature was an independent prognostic factor for patients with PCa. Finally, we found that the expression of TK1 was higher in CRPC tissues than in PCa tissues in three GEO datasets. The results indicated that TK1 promotes the growth and metastasis of PCa cells.

**Conclusions:**

We provide evidence for a PMG signature for PCa patients to accurately predict clinical prognosis. TK1 plays crucial roles in the progression of PCa cells and can be used as a potential therapeutic target for CRPC.

## Introduction

In men, prostate cancer (PCa) is the second most frequently diagnosed cancer, with 1.4 million new cases reported globally in 2020 [1]. Almost all advanced PCa patients will eventually progress to castration-resistant prostate cancer (CRPC) after receiving endocrine therapy, and metastatic CRPC (mCRPC) is the predominant cause of patient death [2,3]. However, the molecular mechanisms involved in PCa progression are still unclear. Once CRPC occurs, whether it is non-metastatic CRPC or metastatic CRPC, patient’s quality of life will be significantly reduced [4]. The mechanisms of castration resistance in PCa are diverse and include changes in mainly the androgen receptor (AR) and non-AR signaling pathways [5,6]. Inhibitors of AR, such as androgen depletion therapy (ADT) as well as new drugs, such as apalutamide, enzalutamide, darolutamide, and abiraterone, have been proven to have substantial benefits in clinical trials and practice. Nonetheless, it is challenging to prevent PCa from advancing to CRPC. As a result, it is crucial to identify novel biomarkers that can effectively distinguish CRPC from PCa, thus aiding in the selection of the most appropriate treatment regimen and timing of application.

Metabolic reprogramming is considered a major feature of cancer. Malignant cells rewire metabolic pathways to meet the energy demands required for tumor progression. In complex metabolic pathways, pyrimidine biosynthesis is crucial for maintaining basic cellular functions in organisms [7]. Previous extensive studies have shown that excessive synthesis and consumption of nucleotide triphosphates (NTPs) and their deoxyribonucleotide triphosphate (dNTP) counterparts are common characteristics of tumor cells. The supraphysiological abundance of nucleotides in cells contributes to various aspects of cancer cell behavior, including uncontrolled proliferation, immune evasion, metastasis, and treatment resistance [8]. Dysregulated *de novo* pyrimidine synthesis not only provides a sufficient amount of nucleotides but also affects other metabolic and cell signaling pathways in cancer [9,10]. Moreover, many oncogenic drivers have been demonstrated to upregulate nucleotide biosynthesis, indicating that this phenotype is crucial for cancer initiation and progression downstream of oncogene activation. For example, three recent articles revealed new drug targets for isocitrate dehydrogenase (IDH)-mutated glioblastoma [11], diffuse midline glioma [12], and medulloblastoma [13], indicating that these brain tumor types have altered metabolism to depend on new pyrimidine synthesis. Researchers have demonstrated that the use of clinically approved *de novo* thymidine synthesis inhibitors can increase the sensitivity of triple-negative breast cancer cells to chemotherapy [14]. Therefore, inhibition of pyrimidine metabolism is highly effective, and many drugs targeting pyrimidine metabolism, including CAD inhibitors, DHODH inhibitors, and UMPs inhibitors, have been investigated for the treatment of various cancers [15].

In a prior investigation, we obtained plasma samples from tumor-free controls (TFC), patients with PCa, and patients with CRPC. Subsequently, exosomes were extracted and purified for metabolomic examination [16]. By analyzing the differentially expressed metabolites among the three groups of patients, we found that the differentially expressed metabolites between the TFC and PCa groups, as well as between the PCa and CRPC groups, are enriched in the pyrimidine metabolism pathway. These findings suggest that pyrimidine metabolism has potential clinical value in PCa. However, systematic research on the interaction between PCa and pyrimidine metabolism has been inadequate. In this study, we identified six differentially expressed pyrimidine metabolism-related genes (PMGs) and used them to differentiate PCa patient populations with different clinical prognoses. We detailed the immune and genomic traits of the patients and created a novel independent prognostic indicator, the PMGscore, which could potentially steer personalized treatment approaches for PCa and CRPC. Additionally, through the use of multiple clinical transcriptomic datasets, we found that the pyrimidine metabolism gene thymidine kinase 1 (TK1) is highly expressed in CRPC, and we preliminarily revealed the functional role of TK1 in cells. Our results provide new insights into the relationship between pyrimidine metabolism and PCa progression.

## Materials and methods

### Patients data collection

The expression profiles of genes in prostate cancer (PCa) patients obtained from The Cancer Genome Atlas (TCGA) were utilized as the primary cohort for discovery, whereas the gene expression profiles sourced from the Gene Expression Omnibus (GEO) were utilized as the secondary cohort for validation. Patients lacking complete follow-up information, normal tissue samples, and samples from metastatic sites were eliminated from the analysis. Finally, our dataset includes gene and clinical information from 481 prostate cancer patients in the TCGA database (https://portal.gdc.cancer.gov/) and data from 2014 prostate cancer patients in the GEO database (GSE70768 and GSE70769 [17]) (https://www.ncbi.nlm.nih.gov/geo/). Transcriptome data from patients with CPPC and PCa, including those in the GSE35988 [18], GSE80609 [19], and GSE32269 [20] cohorts from the GEO database, were used to screen for the target gene TK1. The data processing method was consistent with that used in our previously published articles [21]. TCGA and GEO belong to public databases. The patients involved in the database have obtained ethical approval. Users can download relevant data for free for research and publish relevant articles. Our study is based on open datasets, so there are no ethical issues and other conflicts of interest.

### Selection of candidate PMGs

A total of 98 PMGs were extracted from the dataset of genes related to pyrimidine metabolism (KEGG_PYRIMIDINE_METABOLISM), as documented in the Molecular Signatures Database. Somatic mutations in the genes with high mutation frequencies in patients with PCa in the TCGA cohort were described using the maftools R package. ‘Comparison of gene expression levels in TCGA samples from normal and PCa tissues was conducted using the R software package “edgR”. The results were visualized through heatmaps, volcano plots, and circos plots’. The interactions between 15 PMGs were described using network correlation, and their prognostic significance for PCa patients was assessed.

### Consensus clustering analysis

We categorized PCa patients into various subgroups through consensus clustering analysis. Utilizing the ‘ConsensusClusterPlus’ tool in R, we conducted an analysis to identify the most suitable quantity of clusters and patient allocation through implementing agglomerative pam clustering with Euclidean distances. This involved resampling 80% of the specimens for 1000 iterations. By utilizing the empirical cumulative distribution function plot, we established the optimal number of clusters for clustering.

### Differential expression and functional enrichment analyses

The selection criteria for Differentially Expressed Genes (DEGs) were |log2-Fold Change (FC)| > 0.585 and FDR < 0.05, and the ‘limma’ package in R software was used to identify DEGs between different subgroups. To assess the biological functions and signaling pathways enriched by DEGs, we utilized the ‘clusterProfiler’ tool to perform Gene Ontology (GO) annotation and analyze enrichment in Kyoto Encyclopedia of Genes and Genomes (KEGG) pathways.

### Study of etiology based on whole-genome data

To depict visually genes with high mutation rates across different subgroups, we employed the ‘maftools’ package to create waterfall plots. We leveraged the ‘NMF’, ‘BSgenome’, and ‘MutationalPatterns’ packages in R to download the 30 identified oncogenic mutation signatures from the COSMIC database. We compared these signatures with those identified by NMF to determine the mutation characteristics of PCa.

### Prediction of the treatment response to ICI therapy

To predict how prostate cancer patients will respond to Immune checkpoint inhibitor (ICI) treatment, we utilized the Tumor Immune Dysfunction and Exclusion (TIDE) analysis. A variety of immune features, including immune checkpoint therapy, immunotherapy response, mutation signatures, and tumor mutation burden (TMB), were incorporated.

### Construction of the PMGs-related predictive signature

A prognostic scoring system (PMGscore) was established in this study. The PMGscore was assessed for the selected genes as follows: PMGscore = h0(t) * exp (expression of CANT1 * corresponding CANT1 coefficient + expression of DPYS * corresponding DPYS coefficient + expression of ENTPD5 * corresponding ENTPD5 coefficient + expression of POLR2H * corresponding POLR2H coefficient + expression of RRM2 * corresponding RRM2 coefficient + expression of TK1 * corresponding TK1 coefficient). Patients were divided into low PMGscore and high PMGscore groups based on the median PMGscore.

### Evaluation of the clinical significance of the predictive signature

To investigate the discrepancy in survival rates between the two patient groups, Kaplan-Meier analysis was carried out. Furthermore, the R package ‘survivalROC’ was utilized to generate the Receiver Operating Characteristic (ROC) curve, which evaluates the predictive ability of the PMGscore model. Additionally, prostate cancer patients were categorized based on clinical characteristics to explore the potential of these factors in predicting prognosis.

### Cell culture and transfection

The below prostate cancer cell lines were acquired from the Stem Cell Bank at the Chinese Academy of Sciences: PC3, DU145, C4-2B, 22Rv1, LNCaP, and the human immortalized prostate epithelial cell line RWPE-1. These cancer cells were cultured in RPMI 1640 medium supplemented with 10% fetal bovine serum (FBS-S500, NEWZERUM). The culture medium for RWPE-1 cells is a serum-free keratinocyte medium (catalog number 10744-019, Gibco) containing 5 ng/mL epidermal growth factor (catalog number 10450-013, Gibco). All cell lines were kept in 5% CO_2_ at 37 °C. To knock down TK1, we used small interfering RNA (siRNA) targeting TK1 and a non-specific negative control (NC) siRNA synthesized by RiboBio (Guangzhou, China). The siRNA sequences that specifically target TK1 are shown below: si-h-TK1: ACAAGTGCCTGGTGATCAA.

### RNA extraction and quantitative real-time PCR (qRT-PCR) assays

The main experimental steps were the same as those in the previous study [22]. The primers used in the study are as follows: TK1-forward primer: GGGCAGATCCAGGTGATTCTC; TK1-reverse primer: TGTAGCGAGTGTCTTTGGCATA; GAPDH-forward primer: CAGTCAGCCGCATCTTCTT; and GAPDH-reverse primer: GACAAGCTTCCCGTTCTCAG.

### Cell proliferation

The Cell Counting Kit-8 (CCK-8) (FD3788, Fude) was employed to assess cell viability according to the manufacturer’s guidelines as stated previously [22].

### Colony formation

For colony formation assay, it was conducted as described previously [23].

### Cell cycle analysis

As for cell cycle analysis, it was carried out as stated before [22].

### 5-Ethynyl-2′-deoxyuridine (EdU) incorporation assay

Edu assay was conducted according to the manufacturer’s construction.

### Transwell migration and invasion assay

We assessed cell migration capacity by using Transwell inserts (Costar, Corning, Cambridge, MA, USA) featuring an 8.0 μm pore size chamber. The lower chamber of the insert received an initial addition of 800 μL of complete culture medium. Then, 300 microliters of culture medium without serum with 3 × 10^4^ cells were introduced into the upper section of the insert. The insert was then incubated for a suitable duration (12 h for DU145 cells and 48 h for PC3 cells) before fixing the cells in the upper chamber with 4% paraformaldehyde and staining with Giemsa. Subsequently, the cells on the upper chamber surface of the membrane were removed and imaged using an inverted microscope. At least three fields of view were randomly chosen for imaging, and the cell count was conducted using ImageJ software. For the invasion assay, the transwell membrane in the upper chamber was pre-coated with Matrigel matrix (40111ES08; Easybio Biotechnology Co., Ltd., China) to evaluate the invasive potential of cells. The subsequent steps were identical to those in the migration assay.

### Wound healing assay

An ample number of PCa cells were distributed in a 6-well dish until reaching a cell density of 90%. A series of linear incisions were created using a plastic pipette tip with a volume of 10 μl. Any remaining debris from the cells was rinsed off using PBS, and serum-free culture medium was added for incubation. At 0 and 48 h, the wounds were observed and photographed at the same location using an inverted microscope (Olympus IX71). At least 3 positions were recorded in each group. The wound area was measured using ImageJ software, and the percentage of area recovery was calculated.

### Statistical analysis

Statistical analysis was conducted using GraphPad Prism 9.1.0 (GraphPad Software, La Jolla, CA, USA), SPSS 25.0 (SPSS Company in Chicago, IL, USA), and R4.1.2 software. The two-tailed Student’s *t*-test was applied for data analysis between two groups with normally distributed data. The Mann–Whitney test was utilized for non-normally distributed data. One-way analysis of variance was performed for continuous variables. Associations between survival outcomes and different groups were assessed using log-rank tests. A two-tailed *p*-value < 0.05 indicated statistical significance, and hazard ratios (HRs) along with 95% confidence intervals (CIs) were reported when necessary.

## Results

### Identification and characterization of PMGs involved in PCa progression

**Figure 1.**
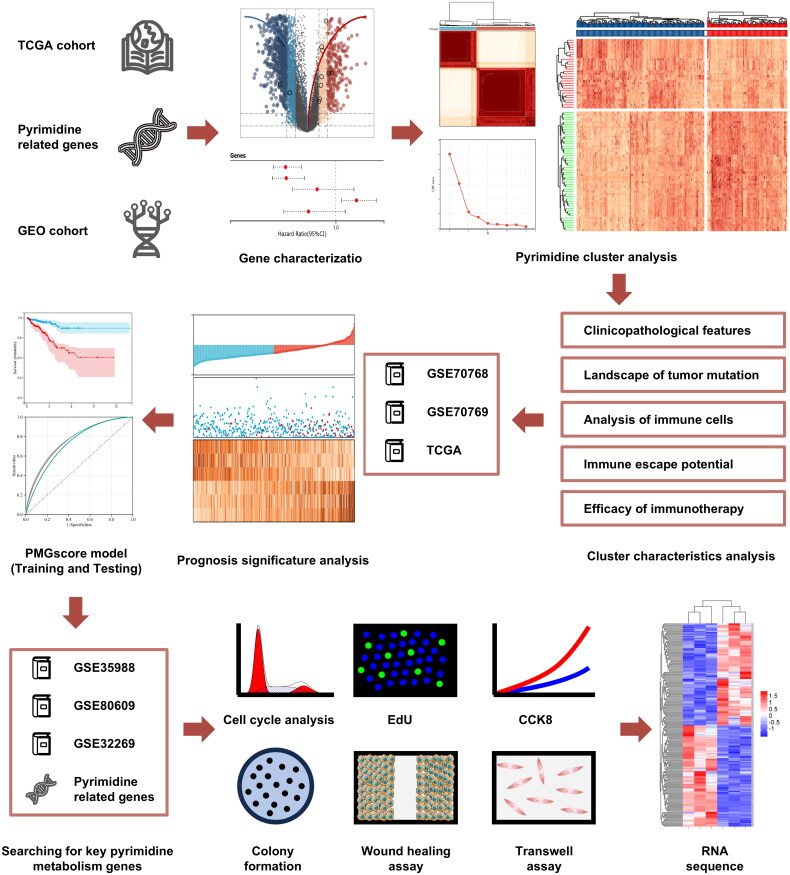
Workflow diagram of this study.

**Figure 2.**
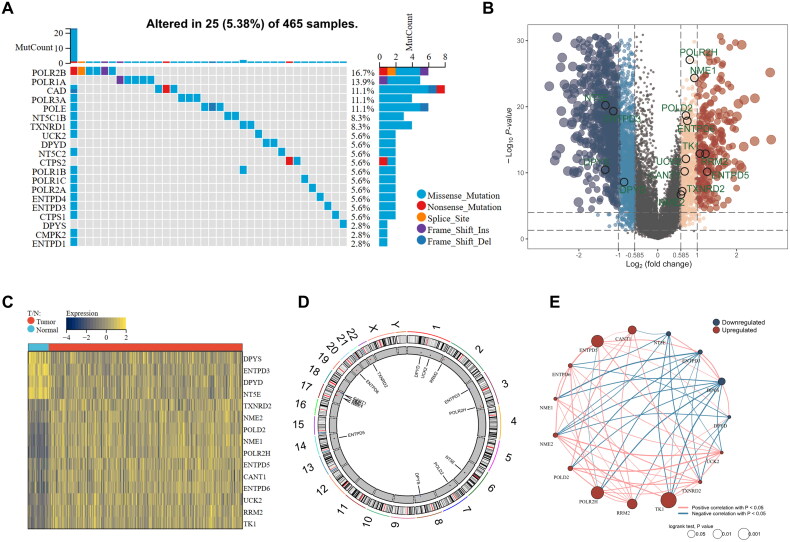
Identification of PMGs and detection of mutations in PCa. (A) Waterfall plot displays the mutational landscape of the 98 PMGs. (B,C) Volcano plot and heatmap of differentially expressed PMGs identified among normal and PCa samples in TCGA database. (D) CNV locations of 15 PMGs are labeled on the chromosome. (E) Interactions among 15 PMGs in PCa. The circle size represents the effect of each regulator on prognosis, the line segment represents the correlation between two genes, and red and blue represent up-regulated and down-regulated expression in prostate tumor tissue, respectively.

The workflow of this study is illustrated in Figure 1. Comprehensive analysis of PMGs in the TCGA cohort was conducted using multigroup data. The top 20 genes with mutation frequencies among the 98 PMGs are shown in Figure 2A. Among them, POLR2B (16.7%) was the gene with the highest mutation frequency, followed by POLR1A (13.9%), CAD (11.1%), POLR3A (11.1%), and POLE (11.1%). We subsequently investigated the expression of the 98 PMGs in the TCGA cohort. By conducting a differential analysis of transcriptome data, we discovered that 15 of the 98 PMGs demonstrated upregulation or downregulation in PCa. The findings are visualized in the volcano plot of Figure 2B and the heatmap of Figure 2C. Figure 2D illustrates the chromosomal locations of the 15 PMGs with copy number variations (CNVs). Figure 2E displays the comprehensive interaction landscape of the 15 PMGs and their prognostic significance for PCa patients. Among these, only DPYS was identified as a protective factor with a risk ratio (HR) <1, while TK1, RRM2, POLR2H, ENTPD5, and CANT1 were considered risk factors. These results reveal the significant heterogeneity in the mutation status and expression of PMG between normal prostate tissues and PCa tissues. Therefore, alterations in PMG expression could play a vital role in the occurrence and development of PCa.

### Construction and clinical significance analysis of molecular classification

**Figure 3.**
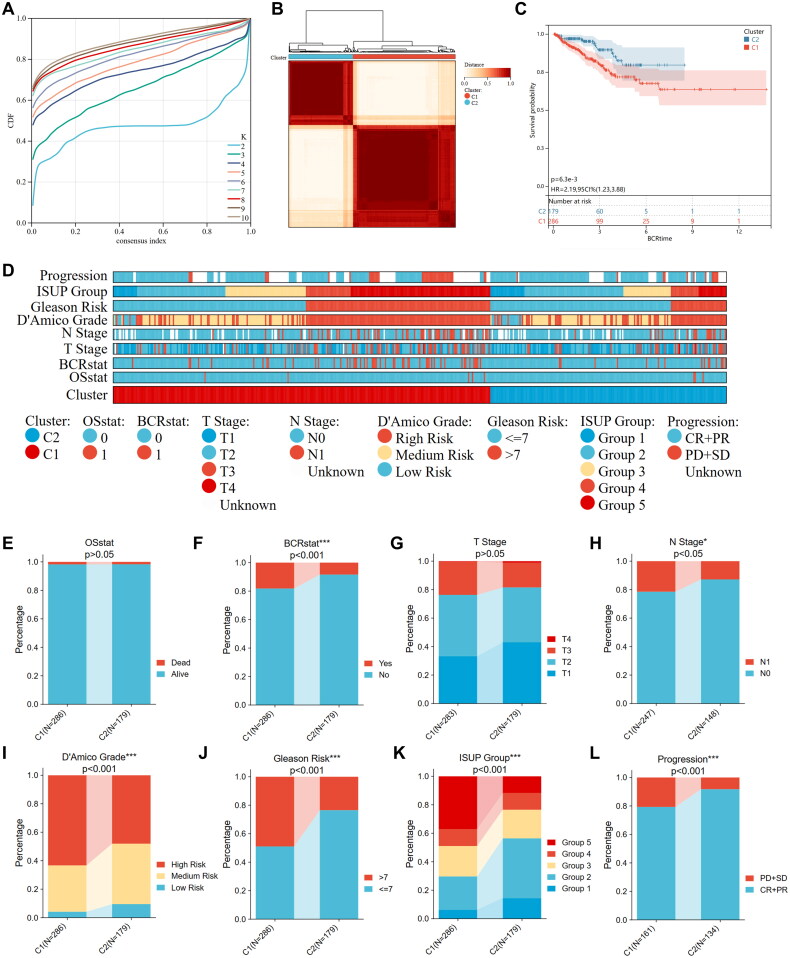
Identification of two clusters with distinct survival outcomes. (A) Consensus clustering matrix for *k* = 2, which was the optimal cluster number. (B) CDF curves of the consensus score from *k* = 2 to 10. (C) Kaplan-Meier curves of the overall survival time of cluster C1 and cluster C2. (D) Heatmap of clinical characteristics among the two clusters. (E–L) Comparisons of OS, BCR, T stage, N stage (*p* < 0.05), Gleason Risk, D’Amico Grade, ISUP Group, and progression between two clusters. **p < 0.01, ***p < 0.001.

By utilizing the 6-PMG prognostic related panel, we performed molecular classification using the CNMF algorithm, which identified two distinct PCa molecular clusters: the C1 cluster (*n* = 286 samples) and the C2 cluster (*n* = 179 samples) (Figures 3A,B). The C2 cluster showed a superior survival probability compared to the C1 cluster (log rank *p* = 6.3e-3) (Figure 3C). In addition, our results demonstrated that the percentages of biochemical recurrence-free status (*p* < 0.001), N stage (*p* < 0.05), Gleason risk (*p* < 0.001), D’Amico grade (*p* < 0.001), ISUP group (*p* < 0.001), and progression (*p* < 0.001) significantly differed between the two clusters (Figures 3D–L).

### Differences in biological mechanisms and genomic features between the two clusters

**Figure 4.**
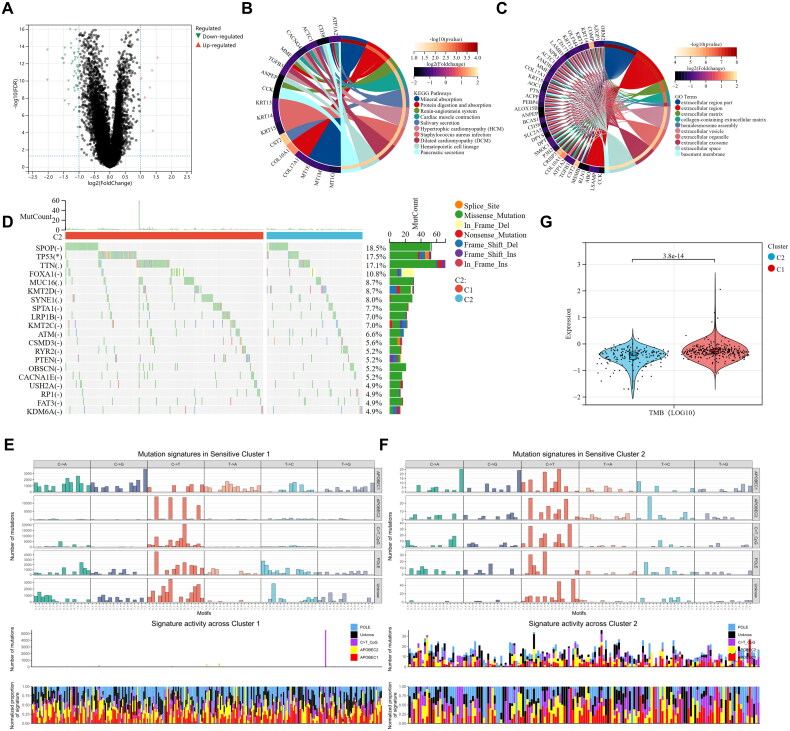
Somatic mutations, mutational signatures, and related signaling pathways of two clusters. (A) Differentially expressed genes between two clusters are shown in a volcano plot. (B,C) GO and KEGG analysis of robust IRGPS. (D) Waterfall plots revealing the somatic mutation distributions of the 20 genes with the highest mutation frequencies in cluster 1 and cluster 2. (E,F) Upper panel, Bayesian NMF was used to identify five mutational signatures in Cluster 1 and Cluster 2. The y-axis shows the number of mutations of each type in each specific sequence. The middle and lower panels show the total number of mutations associated with five mutational signatures (middle) and relative proportion of mutation types (lower panels) in each cluster. (G) The comparisons of TMB between cluster 1 and cluster 2.

To explore the distinctions in biological mechanisms between the two clusters, differential expression analysis was conducted. Eighty differentially expressed genes (DEGs) were identified, out of which 7 genes were significantly upregulated and 73 genes were significantly downregulated (Figure 4A). Next, KEGG and GO enrichment analyses were conducted on the DEGs, resulting in the visualization of several pathways and biological processes (Figures 4B,C). The enriched KEGG pathways were related mainly to mineral absorption, protein digestion and absorption, the renin-angiotensin system, cardiac muscle contraction, salivary secretion, hypertrophic cardiomyopathy (HCM), Staphylococcus aureus infection, dilated cardiomyopathy (DCM), the hematopoietic cell lineage, and pancreatic secretion.

The enrichment of the GO terms was primarily focused on the extracellular region, extracellular matrix, collagen-containing extracellular matrix, hemidesmosome assembly, extracellular vesicle, extracellular organelle, extracellular exosome, extracellular space, and basement membrane. We conducted an additional analysis on the distribution of somatic mutations within the two clusters by utilizing genomic data from the TCGA database (Figure 4D). The mutation frequencies of SPOP, TP53, TTN, etc., in the C2 cluster were greater than those in the C1 cluster. To describe thoroughly the genomic feature landscape, we identified five mutational signatures for the two subtypes. POLE predominated in Cluster 1, whereas APOBEC2 was the main pattern in Cluster 2 (Figures 4E,F). The TMB is a vital biomarker for predicting immunotherapy efficacy. The use of TMB in immunotherapy has attracted much attention. Based on somatic mutation data from the TCGA datasets, we calculated and compared the TMB between the two groups. The findings indicated that the TMB level in C1 was notably higher than in C2 (Figure 4G).

### Mechanisms underlying the immunotherapy response in patients with different clusters

**Figure 5.**
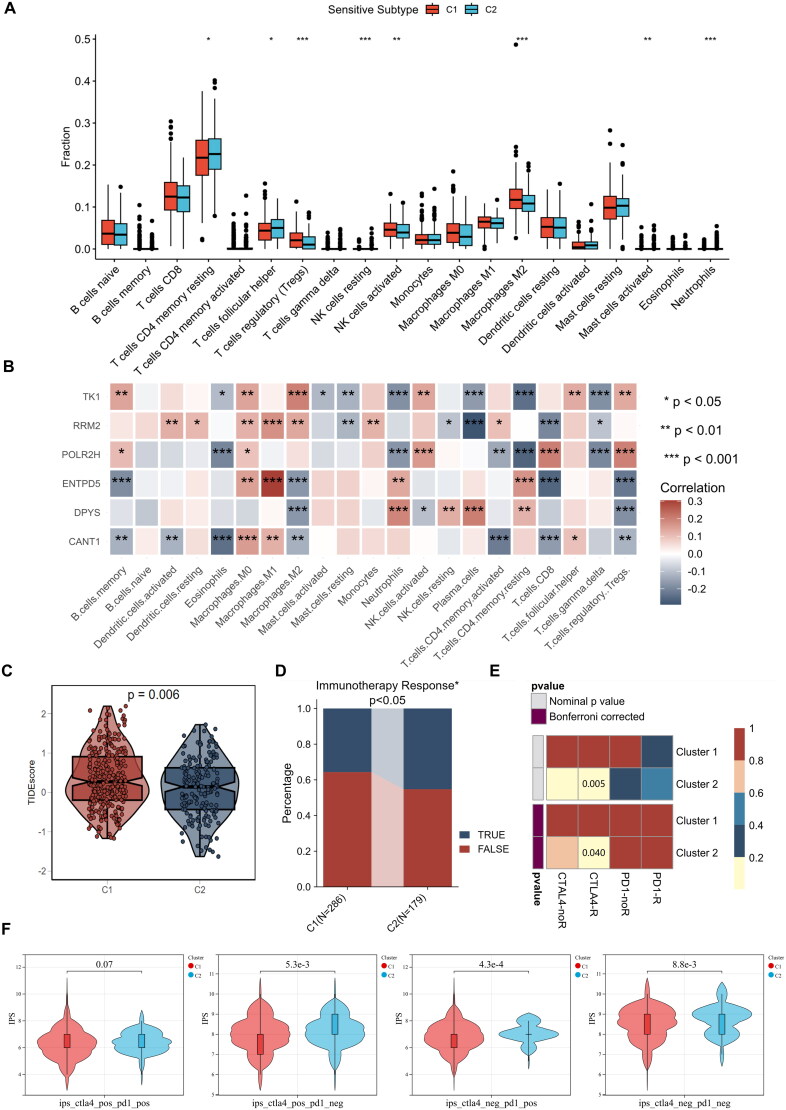
Distinct immunogenomic patterns of two clusters lead to different sensitivity to immunotherapy. (A) Comparisons of the abundances of 21 immune cells in two clusters. (B) Heat map of the correlation between 21 immune cells and key genes in pyrimidine metabolism, with positive correlation in red and negative correlation in blue. (C–E) Comparisons of TIDE score and immunotherapy response between cluster 1 and cluster 2. (F) Subclass mapping analysis for predicting the likelihood of response to ICI therapy of different clusters.

Prior studies have shown that immune cells play a role in helping tumors evade immune responses by communicating and changing how immune cells are situated within the TME. To compare the proportions of 20 different types of immune cells between the two groups, the CIBERSORT algorithm was used. In cluster C1, there were higher levels of regulatory T cells, activated NK cells, and M2 macrophages. Conversely, cluster C2 exhibited increased levels of T cells CD4^+^ memory resting, T cells follicular helper, resting NK cells, activated mast cells, and neutrophils (Figure 5A). Prior studies have shown that patients with PCa who have elevated levels of M2 macrophage infiltration do not respond as effectively to immunotherapy. This could imply an enhanced immunosuppressive capacity and heightened immune evasion potential within cluster C1. In addition, correlations between the six key genes involved in pyrimidine metabolism and various tumor-infiltrating immune cells were analyzed, and a strong correlation was detected between immune infiltrating cells and pyrimidine metabolism genes, especially between M2-polarized macrophages and regulatory cells. These findings suggested that the key genes involved in pyrimidine metabolism may be strongly correlated with immune regulation (*p* < 0.05, |*r*| > 0.2) (Figure 5B). Moreover, there were significant differences in the infiltration of M2 macrophages and regulatory T cells among the different subtypes mentioned above, suggesting that the two different pyrimidine metabolic subtypes may have different responses to immunotherapy. Therefore, we further evaluated the immunotherapy response of the two subtypes through submap mapping and the tumor immune dysfunction and exclusion (TIDE) score.

The TIDE algorithm was employed to assess the immune evasion capacity of individuals with PCa. A higher TIDE score signifies a greater likelihood of immune evasion, and elevated TIDE scores are typically linked to inferior prognostic outcomes, akin to previous biochemical recurrence-free survival results. We showed that the TIDE score of cluster C1 was notably higher than that of cluster C2 (Figure 5C). Moreover, we observed that the proportion of respondents in cluster C2 was notably larger than in cluster C1 (Figures 5D,E). Notably, the PCa patients in cluster C2 were more sensitive to CTLA-4 inhibitors alone (*p* = 5.3e-3), PD-1 inhibitors alone (*p* = 4.3e-4), or no immune checkpoint inhibitors than were those in cluster C1 (*p* = 8.8e-3). However, there was no difference in the combination of PD-1 or CTLA-4 inhibitors between the two clusters (*p* = 0.07) (Figure 5F). These findings suggest that the subtypes of PMG may have the ability to predict, to a certain degree, whether individuals with PCa are likely to respond to immunotherapy.

### Construction and validation of the prognostic signature based on the PMGs

**Figure 6.**
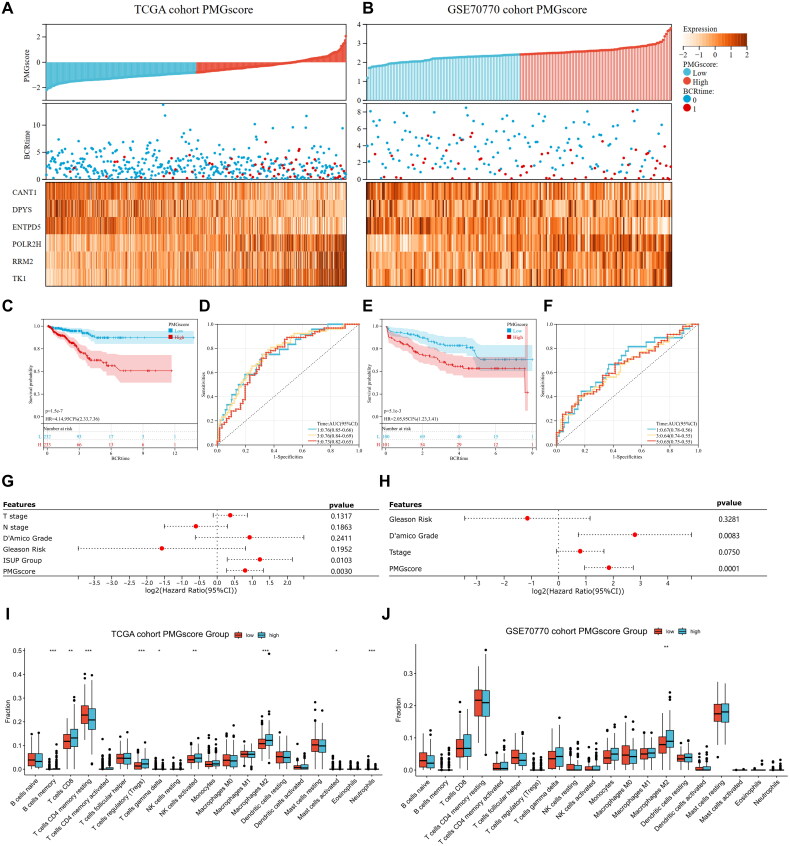
Construction and validation of the PMGs risk signature. (A,B) Survival status, PMG score curves, and heatmap of PMGs expression in the TCGA and GSE70770 cohort. (C) Kaplan-Meier curves for overall survival in TCGA. (D) ROC curves were used to predict the 1, 3, and 5-year survival of patients in TCGA. (E) Kaplan-Meier curves for overall survival in GSE70770. (F) ROC curves were used to predict the 1, 3, and 5-year survival of patients in GSE70770. (G,H) Univariate Cox analyses evaluated the independent prognostic value of PMGs risk signature in PCa patients. (I,J) CIBERSORT analysis of the abundance of 22 tumor-infiltrating immune cells infiltration between the low-PMGscore and high-PMGscore groups.

Six PMGs, namely, TK1, RRM2, POLR2H, ENTPD5, DPYS, and CANT1, were identified and used to construct a prognostic signature. The PCa patients from the TCGA training and validation sets (GSE70770) were individually classified into high- and low-PMG score subgroups based on the median PMG score, to further evaluate the predictive accuracy for PCa (Figures 6A,B). As expected, the death toll among PCa patients increased as the PMG score rose in all groups. The KM survival analysis showed that patients with high-PMG scores had a shorter overall survival (OS) compared to those with low PMG scores. The area under the curve (AUC) validation confirmed that the PMG model served as a statistically significant diagnostic tool for estimating the likelihood of survival in PCa patients (Figures 6C,D). Moreover, the predictive accuracy of the PMG score for PCa was again validated in the GSE70770 cohort (Figures 6E,F). Furthermore, several clinical factors, such as T stage, N stage, D’Amico grade, Gleason risk, the PMG score, and the ISUP Group, are important factors for determining the prognosis of PCa patients. Thus, we explored whether T stage, N stage, D’Amico grade, Gleason risk, the PMG score, and the ISUP grade were independent predictors of PCa prognosis. The findings showed that the PMG score acted as an independent predictor of prognosis for patients suffering from PCa (Figures 6G,H). Furthermore, to delve deeper into the disparities in immune cell infiltration within the TME of PCa patients from different PMG score groups, the CIBERSORT algorithm was utilized. The results demonstrated that the M2 macrophage population was markedly elevated in the high-PMG score subgroup, while the memory resting CD4^+^ T-cell population was considerably increased in the low-PMG score subgroup in both the TCGA and GSE70770 cohorts (Figures 6I,J). Collectively, the aforementioned discoveries reveal a significant correlation and intricate a relationship between the TME and the PMGs, calling for further comprehensive exploration.

### *TK1 is upregulated in PCa cells and accelerates PCa cell proliferation* in vitro

**Figure 7.**
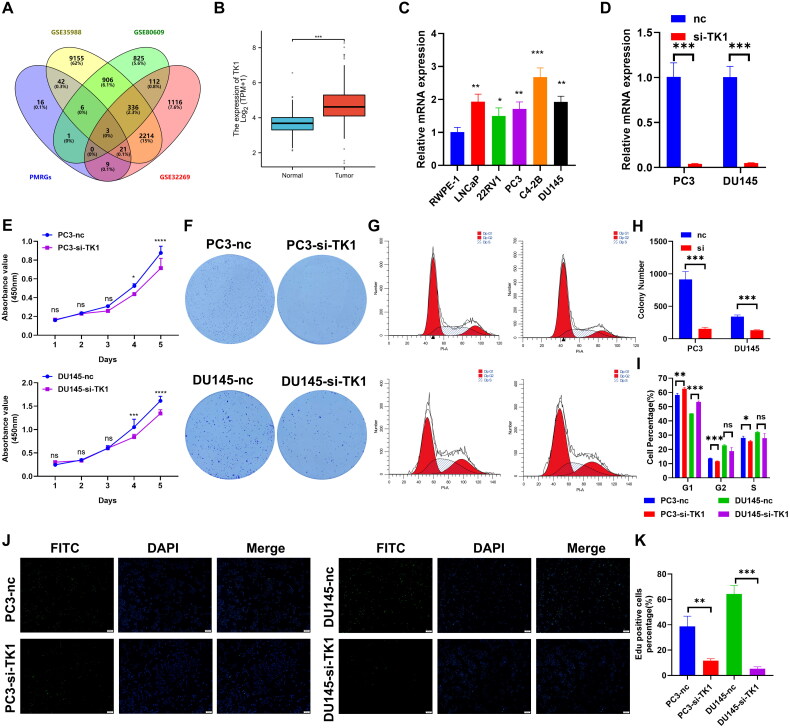
TK1 Accelerates PCa cell proliferation *in vitro*. (A) Venn diagram illustrating overlapping of GEO datasets and PMGs. (B) TK1 is overexpressed in TCGA PCa data. (C) The expression of TK1 in PCa lines was detected by qRT-PCR. (D) The efficiency of knockdown of TK1 in DU145 and PC3 cells was determined by qRT-PCR. (E) PCa cell lines with TK1 knockdown were tested for proliferation using the CCK-8 proliferation assay. (F,H) Colony-formation assay was used for detecting the proliferation ability in TK1-knockdown PCa cell lines. (G,I) Cell-cycle analysis revealed that knocking down TK1 expression in DU145 and PC3 cells increased the percentage of cells in the G1 phase. (J,K) Representative micrographs and quantification of EdU incorporation. Data are presented as means ± standard deviation.

To determine the crucial role of PMGs in differentiating between PCa and CRPC, we selected three published GEO datasets (GSE35988, GSE80609, and GSE32269) for analysis. Through Venn analysis and prognostic factor information from TCGA patients, we screened TK1 as the final investigational target (Figure 7A). To determine the expression of TK1 in PCa, we analyzed the expression data of TK1 in the TCGA database using bioinformatics methods. The results showed that the expression level of TK1 was significantly upregulated in PCa tissues compared to that in normal prostate tissues (Figure 7B). To confirm the essentiality of TK1 for PCa oncogenesis, we initially assessed the expression of endogenous TK1 by Western blot in RWPE-1, DU145, PC3, LNCaP, 22Rv1, and C4-2B cells. The expression level of TK1 in the RWPE-1 cell line was lower than that in the other tested PCa cell lines (Figure 7C).

We selected DU145 and PC3 cells for subsequent studies based on the expression of the cell lines. First, siRNA targeting TK1 was designed to inhibit the expression of these genes in DU145 and PC3 cells. QRT–PCR analyses revealed that, compared with the control, the siRNA significantly silenced intracellular TK1 RNA (Figure 7D). CCK-8 assays showed that proliferation was significantly reduced in TK1-knockdown cells, and colony formation was significantly inhibited in the knockdown group of cells compared with that in the control group (Figures 7E,F,H). The progression of the cell cycle is inextricably linked to the ability of cells to proliferate; therefore, we subsequently used flow cytometry to detect changes in cell cycle distribution. The results showed that knockdown of TK1 in DU145 and PC3 cells induced G1 arrest (Figures 7G,I). Immunofluorescence staining for EdU incorporation assays demonstrated that inhibiting TK1 in DU145 and PC3 cells markedly repressed DNA synthesis. In summary, these findings imply that TK1 promotes cell growth, at least in part, by triggering the G1/S transition in PCa cells (Figures 7J,K).

### *TK1 accelerates PCa cell migration and invasion* in vitro

**Figure 8.**
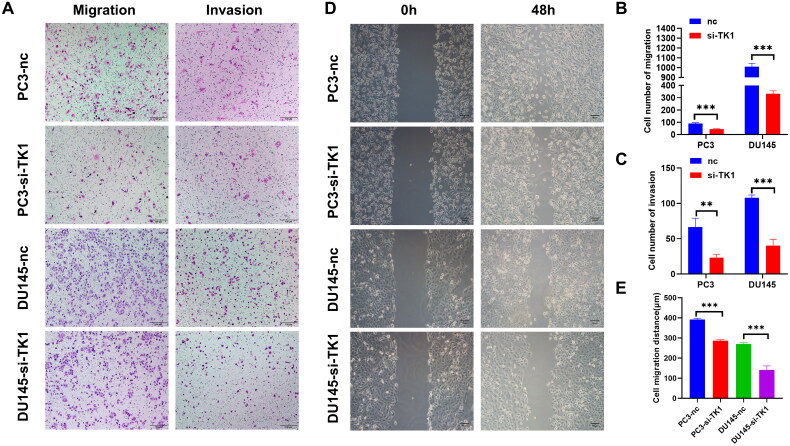
TK1 Accelerates PCa cell migration and invasion *in vitro*. (A–E) TK1 knockdown markedly attenuated cell migration and invasion in DU145 and PC3 cells as measured by transwell migration and wound healing assays. The invasive capability was determined by using matrigel invasion chambers. ns: not significant; *p < 0.05, **p < 0.01, ***p < 0.001. Data are presented as means ± standard deviation.

To determine the potential role of TK1 in promoting PCa cell migration and invasion, transwell, and wound healing assays were performed. The results of migration assays showed that knockdown of TK1 inhibited the migration and invasion of DU145 and PC3 cells (Figures 8A–C). Wound healing experiments yielded the same results (Figures 8D,E). These findings indicated that TK1 accelerates the invasion and metastasis of PCa cells.

### Identification of molecules associated with the underlying mechanisms of TK1

**Figure 9.**
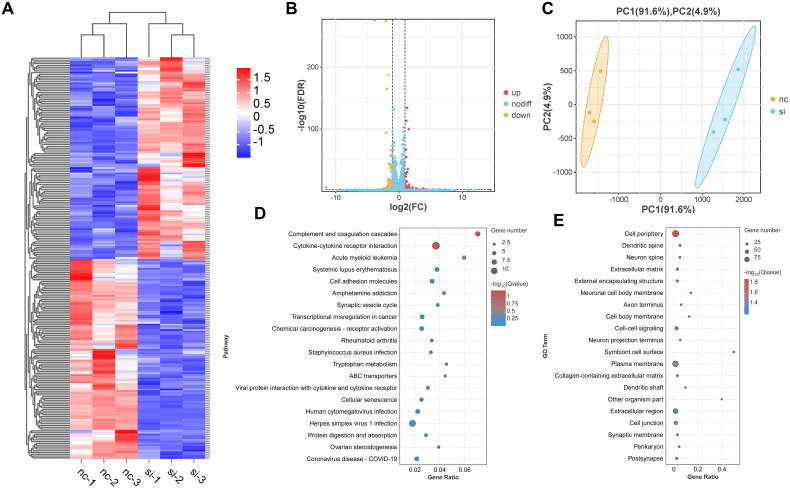
Detection of downstream signal passway of TK1. (A,B) Volcano plot and heatmap of mRNA expression changes between si-nc and si-TK1 groups. (C) PCA analysis of RNA-seq data from nc and si-TK1 groups (*n* = 3 per group). (D,E) Top 20 of KEGG pathway and GO enrichment of differential genes in si-nc and si-TK1 groups are shown by bubble plots.

To explore the potential genes associated with TK1 in PCa cells, RNA transcriptome sequencing was performed on PC3/nc or PC3/si-TK1 cells. Following the silencing of TK1, a total of 112 genes demonstrated an expression change of over 2-fold, while 111 genes showed a reduction in abundance (< −2-fold change) (Figures 9A–C). KEGG pathway analysis and GO analysis of the differentially expressed genes demonstrated that TK1 knockdown can affect the biological functions of PCa cells, including cell adhesion, cellular senescence, and cell junctions, which are critical for tumor progression (Figures 9D,E).

## Discussion

The progression of PCa to CRPC and subsequent metastasis are the main causes of death [24]. Due to the natural or acquired resistance of CRPC, its complex resistance mechanism makes clinical treatment of CRPC very difficult. As the growth of PCa depends on AR signaling, the primary treatment for PCa is ADT. Although ADT is initially effective, most tumors tend to advance to CRPC even when the most potent anti-androgen medications are employed. At present, there is no available curative treatment. It is widely accepted that the reactivation of AR signaling propels the progression of CRPC *via* multiple mechanisms, including AR gene amplification/mutation [18,25], the generation of AR splicing variants [26], AR protein transportation [27], post-translational modification of AR [28], abnormal androgen synthesis [29], abnormal expression of AR coregulators [30], and alternative activation of AR through cytokines and growth factors in the absence of androgens [31]. Therefore, potential therapeutic approaches for PCa have always been a major focus of research.

In recent years, metabolic reprogramming has been recognized as a new marker of tumorigenesis. Tumors regulate metabolic processes during development by synthesizing the energy needed for survival while providing the driving force for tumor cell proliferation, invasion, and metastasis [32]. The energy metabolism of tumors determines their fate. In PCa, metabolic pathways differ markedly from those in normal prostate epithelial cells and exhibit significant heterogeneity at different disease stages. Studies have reported that metabolic reprogramming of glucose [33], lipid [34,35], and amino acid metabolism [36] is an important energy characteristic that drives the formation of CRPC. Pyrimidine is a crucial compound necessary for nucleic acid, phospholipid, glucose metabolism, and protein glycosylation. In all living organisms, pyrimidine biosynthesis is preserved during the intricate metabolic processes, playing a critical role in sustaining cellular activities like DNA and RNA synthesis [37]. Numerous studies have demonstrated a close connection between pyrimidine metabolism dysfunction and cancer progression. Consequently, many drugs that target pyrimidine metabolism have been approved for treating various types of cancer [38,39]. Mounting research has revealed the interplay between oncogenic signal transduction and pyrimidine synthesis in tumorigenesis. KRAS mutant [40], PTEN deficient [41], and p53 deficient [42] cells display an elevated pyrimidine *de novo* synthesis flux. Relying on the pyrimidine pathway results in the discovery of potential synthetic lethal targets in pyrimidine synthesis within these cells, highlighting the metabolic weaknesses of different functionally altered mutant cancers.

In our previous research, we uncovered the potential importance of pyrimidine metabolism in PCa by observing metabolic differences in plasma exosomes from patients with TFC, PCa, and CRPC [16]. Building on this, our current study aimed to assess the potential function of pyrimidine metabolism in PCa, offering prognostic predictions and therapeutic recommendations for patients with PCa. Our study’s results have important implications for predicting the outcomes of patients with PCa in clinical practice. The CNMF algorithm was utilized to detect unique molecular groups using PMGs. We observed that the survival probability of patients in cluster C2 was superior to that of patients in cluster C1. Notably, cluster C1 was characterized by elevated expression levels of regulatory T cells and M2 macrophages. This finding may explain why the prognosis of patients in cluster C2 was more favorable compared to those in cluster C1.

The treatment of PCa with immunotherapy, which includes checkpoint inhibitors, cytokines, and therapeutic cancer vaccines, has garnered significant attention [43]. Currently, monoclonal antibodies targeting PD1/PDL1 and CTLA4 are used to treat metastatic castration-resistant prostate cancer (mCRPC) patients, but the trial outcomes have been underwhelming. Only a small subset of patients benefits from immunotherapy, and explaining this heterogeneity remains a challenge [44,45]. This untargeted approach may contribute to the shortcomings of immunotherapy clinical trials. Based on our previous clinical sequencing data, we initially targeted the pyrimidine metabolism pathway. Therefore, this study explored the relationship between pyrimidine metabolism and prostate cancer immunotherapy efficacy. The results showed that cluster C1 had a higher TIDE score and a better response to immunotherapy than cluster C2. In addition, we generated a classifier based on PMG score for use in molecular classification prediction, promoting clinical application. To the best of our knowledge, this study is the first to analyze the clinical significance and differences in the expression of immune genes related to pyrimidine metabolism. Finally, we developed a model based on six PMGs (TK1, RRM2, POLR2H, ENTPD5, DPYS, and CANT1), with significant differences in expression and prognostic value.

To determine the crucial role of pyrimidine metabolism genes in the progression of CRPC, we selected three published GEO datasets for analysis. Through Venn analysis and prognostic factor information from TCGA patients, we screened TK1 as the final investigational target. TK1 is an enzyme associated with DNA repair pathways that aid in thymidine renewal, DNA construction, and resolution of DNA damage [46]. Thymidine enables the migration of substances from the extracellular environment to the cell membrane, where it is converted into its monophosphate form (dTMP) by TK1 within the cytoplasm. Before DNA replication, enzymes in the cytoplasm transform dTMP into its triphosphate version, deoxythymidine triphosphate (dTTP). Nucleotides are transported into the cell nucleus through the nuclear pore complex for DNA replication and transcription. While the *de novo* pathway offers another method for nucleotide production, it is mainly anabolic and less efficient in terms of cellular energy conservation. During *de novo* synthesis, deoxyuridine monophosphate (dUMP) is converted to dTMP by thymidylate synthase with the assistance of folic acid and vitamin B12.

Due to its lower energy requirements, the rescue pathway is the preferred route within cells. As there are two methods for producing dTTP, TK1 is not absolutely essential for cell survival. Since 1960, scientists have discovered heightened amounts of TK1 in the blood of individuals afflicted with various forms of cancer, such as lung, colon, breast, and prostate cancer [47]. In hepatocellular carcinoma, TK1 drives tumor progression in an enzyme-dependent and -independent manner [48]. TK1 mediates the synergistic antitumor effects of ubenimex and celecoxib through regulation of the colorectal cancer cell cycle [49]. TK1 drives the malignant progression of skin melanoma and metabolic reprogramming [50]. Disruption of TK1 suppresses the growth, invasion, and migration of thyroid cancer cells, as well as the induction of EMT [51]. The lack of TK1 hinders the progression of lung cancer by decreasing GDF15 expression and metastatic capabilities [52]. However, it is unclear whether TK1 promotes PCa progression and CRPC progression. To elucidate the key role of TK1 in PCa, we selected PC3 and DU145 cells with high TK1 expression for functional loss experiments. Suppressing TK1 expression in PC3 and DU145 cells markedly inhibited cell growth, migration, and invasion. These findings emphasize the vital function of TK1 in PCa development and metastasis. To analyze the mechanism by which TK1 promotes PCa proliferation, migration, and invasion, we performed transcriptome sequencing and found that TK1 regulates cell adhesion, cellular senescence, and cell junctions, which are critical for tumor proliferation, migration, and invasion. These results indicate that TK1 plays a key role in PCa progression.

There are limitations in our research. First, we have not yet collected tissue from PCa and CRPC patients for the detection of TK1 protein expression. Additionally, we have not yet explored whether TK1 can promote resistance to ADT in PCa cells. Therefore, the findings of this study require verification through relevant experiments.

## Conclusions

In summary, our study demonstrated that genes associated with different subtypes of pyrimidine metabolism can serve as potent prognostic indicators for prostate cancer and could be potential targets for prostate cancer treatment. TK1 could be a prominent molecular marker to predict PCa progression.

## Data Availability

The TCGA-PRAD dataset used in this study can be obtained from TCGA (https://portal.gdc.cancer.gov/). The GEO datasets for PCa patients include GSE70768, GSE70769, GSE35988, GSE80609, and GSE32269 were downloaded from the GEO database (https://www.ncbi.nlm.nih.gov/geo/). This article was based on a secondary analysis of published literature and data from public databases, and no new data had been generated. The raw data files supporting the findings from our own experiments in this study were available from the corresponding author, Shan-Chao Zhao, upon reasonable request.

## References

[1] Sung H, Ferlay J, Siegel RL, et al. Global Cancer Statistics 2020: GLOBOCAN estimates of incidence and mortality worldwide for 36 cancers in 185 countries. CA Cancer J Clin. 2021;71(3):209–249. doi: 10.3322/caac.21660. 33538338

[2] Bradley CA. Myeloid-derived IL-23 drives CRPC. Nat Rev Urol. 2018;15(9):528. doi: 10.1038/s41585-018-0059-0. 29991724

[3] Sandhu S, Moore CM, Chiong E, et al. Prostate cancer. Lancet. 2021;398(10305):1075–1090. doi: 10.1016/S0140-6736(21)00950-8. 34370973

[4] Miller K. [Review on quality of life in CRPC patients]. Aktuelle Urol. 2017;48(3):219–224. doi: 10.1055/s-0043-100492. 28614882

[5] Gebrael G, Fortuna GG, Sayegh N, et al. Advances in the treatment of metastatic prostate cancer. Trends Cancer. 2023;9(10):840–854. doi: 10.1016/j.trecan.2023.06.009. 37442702

[6] Riley CM, Elwood JML, Henry MC, et al. Current and emerging approaches to noncompetitive AR inhibition. Med Res Rev. 2023;43(5):1701–1747. doi: 10.1002/med.21961. 37062876

[7] Yang C, Zhao Y, Wang L, et al. *De novo* pyrimidine biosynthetic complexes support cancer cell proliferation and ferroptosis defence. Nat Cell Biol. 2023;25(6):836–847. doi: 10.1038/s41556-023-01146-4. 37291265

[8] Mullen NJ, Singh PK. Nucleotide metabolism: a pan-cancer metabolic dependency. Nat Rev Cancer. 2023;23(5):275–294. doi: 10.1038/s41568-023-00557-7. 36973407 PMC10041518

[9] Ma H, Cui J, Liu Z, et al. Blockade of *de novo* pyrimidine biosynthesis triggers autophagic degradation of oncoprotein FLT3-ITD in acute myeloid leukemia. Oncogene. 2023;42(45):3331–3343. doi: 10.1038/s41388-023-02848-7. 37752234

[10] He D, Chen M, Chang L, et al. *De novo* pyrimidine synthesis fuels glycolysis and confers chemoresistance in gastric cancer. Cancer Lett. 2022;549:215837. doi: 10.1016/j.canlet.2022.215837. 35921972

[11] Shi DD, Savani MR, Levitt MM, et al. *De novo* pyrimidine synthesis is a targetable vulnerability in IDH mutant glioma. Cancer Cell. 2022;40(9):939–956.e16. doi: 10.1016/j.ccell.2022.07.011. 35985343 PMC9515386

[12] Pal S, Kaplan JP, Nguyen H, et al. A druggable addiction to *de novo* pyrimidine biosynthesis in diffuse midline glioma. Cancer Cell. 2022;40(9):957–972.e10. doi: 10.1016/j.ccell.2022.07.012. 35985342 PMC9575661

[13] Gwynne WD, Suk Y, Custers S, et al. Cancer-selective metabolic vulnerabilities in MYC-amplified medulloblastoma. Cancer Cell. 2022;40(12):1488–1502.e7. doi: 10.1016/j.ccell.2022.10.009. 36368321

[14] Brown KK, Spinelli JB, Asara JM, et al. Adaptive reprogramming of *de novo* pyrimidine synthesis is a metabolic vulnerability in triple-negative breast cancer. Cancer Discov. 2017;7(4):391–399. doi: 10.1158/2159-8290.CD-16-0611. 28255083 PMC5380483

[15] Wang W, Cui J, Ma H, et al. Targeting pyrimidine metabolism in the era of precision cancer medicine. Front Oncol. 2021;11:684961. doi: 10.3389/fonc.2021.684961. 34123854 PMC8194085

[16] Liu P, Wang W, Wang F, et al. Alterations of plasma exosomal proteins and motabolies are associated with the progression of castration-resistant prostate cancer. J Transl Med. 2023;21(1):40. doi: 10.1186/s12967-022-03860-3. 36681849 PMC9867857

[17] Ross-Adams H, Lamb AD, Dunning MJ, et al. Integration of copy number and transcriptomics provides risk stratification in prostate cancer: a discovery and validation cohort study. EBioMedicine. 2015;2(9):1133–1144. doi: 10.1016/j.ebiom.2015.07.017. 26501111 PMC4588396

[18] Grasso CS, Wu Y-M, Robinson DR, et al. The mutational landscape of lethal castration-resistant prostate cancer. Nature. 2012;487(7406):239–243. doi: 10.1038/nature11125. 22722839 PMC3396711

[19] Yun SJ, Kim S-K, Kim J, et al. Transcriptomic features of primary prostate cancer and their prognostic relevance to castration-resistant prostate cancer. Oncotarget. 2017;8(70):114845–114855. doi: 10.18632/oncotarget.22296. 29383125 PMC5777737

[20] Cai C, Wang H, He HH, et al. ERG induces androgen receptor-mediated regulation of SOX9 in prostate cancer. J Clin Invest. 2013;123(3):1109–1122. doi: 10.1172/JCI66666. 23426182 PMC3582143

[21] Xie X, Dou C-X, Luo M-R, et al. Plasma cell subtypes analyzed using artificial intelligence algorithm for predicting biochemical recurrence, immune escape potential, and immunotherapy response of prostate cancer. Front Immunol. 2022;13:946209. doi: 10.3389/fimmu.2022.946209. 36569837 PMC9772552

[22] Yu Y-Z, Lv D-J, Wang C, et al. Hsa_circ_0003258 promotes prostate cancer metastasis by complexing with IGF2BP3 and sponging miR-653-5p. Mol Cancer. 2022;21(1):12. doi: 10.1186/s12943-021-01480-x. 34986849 PMC8729084

[23] Xie T, Fu D-J, Li Z-M, et al. CircSMARCC1 facilitates tumor progression by disrupting the crosstalk between prostate cancer cells and tumor-associated macrophages via miR-1322/CCL20/CCR6 signaling. Mol Cancer. 2022;21(1):173. doi: 10.1186/s12943-022-01630-9. 36045408 PMC9434883

[24] Cai M, Song X-L, Li X-A, et al. Current therapy and drug resistance in metastatic castration-resistant prostate cancer. Drug Resist Updates. 2023;68:100962. doi: 10.1016/j.drup.2023.100962. 37068396

[25] Bubendorf L, Kononen J, Koivisto P, et al. Survey of gene amplifications during prostate cancer progression by high-throughout fluorescence *in situ* hybridization on tissue microarrays. Cancer Res. 1999;59(4):803–806. 10029066

[26] Cattrini C, Rubagotti A, Zinoli L, et al. Role of circulating tumor cells (CTC), androgen receptor full length (AR-FL) and androgen receptor splice variant 7 (AR-V7) in a prospective cohort of castration-resistant metastatic prostate cancer patients. Cancers. 2019;11(9):1365. doi: 10.3390/cancers11091365. 31540293 PMC6770005

[27] Rodriguez-Bravo V, Pippa R, Song W-M, et al. Nuclear pores promote lethal prostate cancer by increasing POM121-driven E2F1, MYC, and AR nuclear import. Cell. 2018;174(5):1200–1215.e20. doi: 10.1016/j.cell.2018.07.015. 30100187 PMC6150493

[28] Gioeli D, Paschal BM. Post-translational modification of the androgen receptor. Mol Cell Endocrinol. 2012;352(1–2):70–78. doi: 10.1016/j.mce.2011.07.004. 21820033

[29] Pernigoni N, Zagato E, Calcinotto A, et al. Commensal bacteria promote endocrine resistance in prostate cancer through androgen biosynthesis. Science. 2021;374(6564):216–224. doi: 10.1126/science.abf8403. 34618582

[30] Wei J, Yin L, Li J, et al. Bidirectional cross-talk between MAOA and AR promotes hormone-dependent and castration-resistant prostate cancer. Cancer Res. 2021;81(16):4275–4289. doi: 10.1158/0008-5472.CAN-21-0198. 34167949 PMC8373824

[31] Calcinotto A, Spataro C, Zagato E, et al. IL-23 secreted by myeloid cells drives castration-resistant prostate cancer. Nature. 2018;559(7714):363–369. doi: 10.1038/s41586-018-0266-0. 29950727 PMC6461206

[32] Martínez-Reyes I, Chandel NS. Cancer metabolism: looking forward. Nat Rev Cancer. 2021;21(10):669–680. doi: 10.1038/s41568-021-00378-6. 34272515

[33] Petrella G, Corsi F, Ciufolini G, et al. Metabolic reprogramming of castration-resistant prostate cancer cells as a response to chemotherapy. Metabolites. 2022;13(1):65. doi: 10.3390/metabo13010065. 36676990 PMC9865398

[34] Liu S, Lai J, Feng Y, et al. Acetyl-CoA carboxylase 1 depletion suppresses *de novo* fatty acid synthesis and mitochondrial β-oxidation in castration-resistant prostate cancer cells. J Biol Chem. 2023;299(1):102720. doi: 10.1016/j.jbc.2022.102720. 36410440 PMC9771725

[35] Chen H, Guo S, Liu Y, et al. A stable NIR fluorescent probe for imaging lipid droplets in triple-negative breast cancer. Sens Actuators B Chem. 2024;398.134740.

[36] Dai X, Shi X, Luo M, et al. Integrative analysis of transcriptomic and metabolomic profiles reveals enhanced arginine metabolism in androgen-independent prostate cancer cells. BMC Cancer. 2023;23(1):1241. doi: 10.1186/s12885-023-11707-3. 38104097 PMC10724921

[37] Löffler M, Fairbanks LD, Zameitat E, et al. Pyrimidine pathways in health and disease. Trends Mol Med. 2005;11(9):430–437. doi: 10.1016/j.molmed.2005.07.003. 16098809

[38] Robinson AD, Eich ML, Varambally S. Dysregulation of *de novo* nucleotide biosynthetic pathway enzymes in cancer and targeting opportunities. Cancer Lett. 2020;470:134–140. doi: 10.1016/j.canlet.2019.11.013. 31733288

[39] Walter M, Herr P. Re-discovery of pyrimidine salvage as target in cancer therapy. Cells. 2022;11(4):739. doi: 10.3390/cells11040739. 35203388 PMC8870348

[40] Koundinya M, Sudhalter J, Courjaud A, et al. Dependence on the pyrimidine biosynthetic enzyme DHODH is a synthetic lethal vulnerability in mutant KRAS-driven cancers. Cell Chem Biol. 2018;25(6):705–717.e11. doi: 10.1016/j.chembiol.2018.03.005. 29628435

[41] Mathur D, Stratikopoulos E, Ozturk S, et al. PTEN regulates glutamine flux to pyrimidine synthesis and sensitivity to dihydroorotate dehydrogenase inhibition. Cancer Discov. 2017;7(4):380–390. doi: 10.1158/2159-8290.CD-16-0612. 28255082 PMC5562025

[42] Ding D, Blee AM, Zhang J III, et al. Gain-of-function mutant p53 together with ERG proto-oncogene drive prostate cancer by beta-catenin activation and pyrimidine synthesis. Nat Commun. 2023;14(1):4671., doi: 10.1038/s41467-023-40352-4. 37537199 PMC10400651

[43] Sridaran D, Bradshaw E, DeSelm C, et al. Prostate cancer immunotherapy: improving clinical outcomes with a multi-pronged approach. Cell Rep Med. 2023;4(10):101199. doi: 10.1016/j.xcrm.2023.101199. 37738978 PMC10591038

[44] do Pazo C, Webster RM. The prostate cancer drug market. Nat Rev Drug Discov. 2021;20(9):663–664. doi: 10.1038/d41573-021-00111-w. 34145436

[45] He Y, Xu W, Xiao YT, et al. Targeting signaling pathways in prostate cancer: mechanisms and clinical trials. Signal Transduct Target Ther. 2022;7(1):198. doi: 10.1038/s41392-022-01042-7. 35750683 PMC9232569

[46] Bitter EE, Townsend MH, Erickson R, et al. Thymidine kinase 1 through the ages: a comprehensive review. Cell Biosci. 2020;10(1):138. doi: 10.1186/s13578-020-00493-1. 33292474 PMC7694900

[47] Jagarlamudi KK, Shaw M. Thymidine kinase 1 as a tumor biomarker: technical advances offer new potential to an old biomarker. Biomark Med. 2018;12(9):1035–1048. doi: 10.2217/bmm-2018-0157. 30039979

[48] Li Q, Zhang L, Yang Q, et al. Thymidine kinase 1 drives hepatocellular carcinoma in enzyme-dependent and -independent manners. Cell Metab. 2023;35(6):912–927.e7. doi: 10.1016/j.cmet.2023.03.017. 37071992

[49] Wang A, Shang Y, Ni J, et al. Thymidine kinase 1 mediates the synergistic antitumor activity of ubenimex and celecoxib via regulation of cell cycle in colorectal cancer. J Pharmacol Exp Ther. 2022;382(2):188–198. doi: 10.1124/jpet.122.001118. 35868865

[50] Zuo S, Wang H, Li L, et al. Thymidine kinase 1 drives skin cutaneous melanoma malignant progression and metabolic reprogramming. Front Oncol. 2022;12:802807. doi: 10.3389/fonc.2022.802807. 35311151 PMC8927676

[51] Liu C, Wang J, Zhao L, et al. Knockdown of thymidine kinase 1 suppresses cell proliferation, invasion, migration, and epithelial-mesenchymal transition in thyroid carcinoma cells. Front Oncol. 2019;9:1475. doi: 10.3389/fonc.2019.01475. 32064235 PMC7000458

[52] Malvi P, Janostiak R, Nagarajan A, et al. Loss of thymidine kinase 1 inhibits lung cancer growth and metastatic attributes by reducing GDF15 expression. PLOS Genet. 2019;15(10):e1008439. doi: 10.1371/journal.pgen.1008439. 31589613 PMC6797230

